# Scaling Scientific Cellular Automata Microstructure Evolution Model of Static Recrystallization toward Practical Industrial Calculations

**DOI:** 10.3390/ma14154082

**Published:** 2021-07-22

**Authors:** Mateusz Sitko, Krzysztof Banaś, Lukasz Madej

**Affiliations:** Department of Applied Computer Science and Modelling, AGH University of Science and Technology, Mickiewicza 30, 30-059 Krakow, Poland; kbanas@agh.edu.pl (K.B.); lmadej@agh.edu.pl (L.M.)

**Keywords:** modeling, metal forming, microstructure, parallelization, cellular automata

## Abstract

An attempt to bridge the gap between capabilities offered by advanced full-field microstructure evolution models based on the cellular automata method and their practical applications to daily industrial technology design was the goal of the work. High-performance parallelization techniques applied to the cellular automata static recrystallization (CA-SRX) model were selected as a case study. Basic assumptions of the CA-SRX model and developed modifications allowing high-performance computing are presented within the paper. Particular attention is placed on the development of the parallel computation scheme allowing numerical simulations even for a large volume of material. The development of new approaches to handle communication within the distributed environment is also addressed in the paper as a means to obtain higher computational efficiency. Evaluation of model limits was based on the scalability analysis. The investigation was carried out for the 3D and 2D case studies. Therefore, the complex static recrystallization cellular automata simulation taking into account the influence of recovery, nucleation based on accumulated energy, and the progress of recrystallization as a function of stored energy and grain boundary mobility with high-performance computing capabilities is now possible. The research highlighted that parallelization is more effective with an increasing number of cellular automata cells processed during the entire simulation. It was also proven that the developed parallelization scheme and communication mechanism provides a possibility of obtaining scaled speedup over 700 times for 2D and over 800 times for 3D computational domains, which is crucial for future applications in industrial practice. Therefore, the presented approach’s main advantage is based on the possibility of running the calculation based on input data obtained directly from high-resolution 3D imaging of the microstructure. With that, the full immersion of the experimental results into the numerical model is possible. The second novelty aspect of this work is related to the identification of the quality of model predictions as a function of model size reductions.

## 1. Introduction

Modern approaches to the production of complex metallic components are increasingly supported by advanced laboratory research [1,2] and the constant development of more and more accurate numerical models [3,4,5]. There are currently two main trends in the use of numerical models by the industry. On one hand, high-speed models are required because they can predict the overall material behavior for an online adaptation of process conditions [6]. As a result, operators, or even control systems, can modify process parameters according to the predicated material state and obtain the best properties of the final products [7]. On the other hand, using a set of experimental data as input for the state-of-the-art numerical modeling techniques is possible during the offline technology design stage. In this case, the most valuable 3D data about the metallic material state can be obtained from serial sectioning [8] or synchrotron tomography [9,10,11]. These data describe significant morphological heterogeneities of the microstructure, but their incorporation into the numerical simulation requires substantial computing spaces and entails further limitations related to the simulation time. Numerical approaches capable of handling such 3D data during the modeling stage are called full-field models and are, most of the time, based on phase field [12], vertex [13], level set [3], Monte Carlo [14], or cellular automata [15] methods. The latter is often used in numerical model microstructure evolution during recrystallization that occurs under or after the deformation [16,17]. These approaches have a rather academic character most of the time due to the mentioned long computational times. Therefore, various attempts to improve the simulation time, starting from limiting the problem size or reducing the number of considered cells during a single iteration, are described in the literature [18,19]. These solutions limit the computational time, but unfortunately affect the prediction quality. For this reason, advanced techniques of calculation parallelization are more often used. Some of the solutions are focused on parallelization for processors within a single computing unit [20], more advanced are those designed for massively parallel computing centers [21] or modern graphics cards [22]. In the former case, the number of working threads is limited to the number of CPU cores (independent processing units that read and execute instructions), which are available on a computing unit. It should also be mentioned that some CPU processors are enhanced by hyper-threading technology, which offers an additional increase in the performance of approximately 30% [23]. When a single computation unit is concerned, the OpenMP technique is most often used to parallel cellular automata simulations (e.g., [24,25,26]). These approaches are dedicated to symmetric multiprocessing (SMP) architectures. The solution provides an efficient way to use a single computational unit with a high number of cores offered by new processors, but it can only use the amount of memory available within this particular unit. This is a significant limitation when simulations of large 3D computational domains generated directly on experimental data are considered. Therefore, approaches based on a distributed memory and the message passing interface (MPI) standard are now intensively applied to CA algorithms [27,28]. In these approaches, multiple computational units called nodes (based mostly on a two CPU platform) are connected within a local area network (LAN) and are used to perform the same set of tasks. Computations realized on such a cluster can be controlled by a middleware software layer that allows an end-user to treat the whole cluster as a single computing unit (e.g., via a single system image concept). In this case, the combination of the OpenMP application programming interface and the MPI standard is used. These approaches provide an efficient way to use supercomputer capabilities for scientific calculations based on the 3D CA method [29,30]. Advantages of these type of solutions were also highlighted in [31], where particularly static recrystallization simulation was considered. The authors pointed out that parallelization is a key factor to speed up microstructure evolution simulation. However, to take full advantage of the possibilities offered by the available computing clusters, more efficient parallelization schemes and communication mechanisms have to be developed. As a result, full field modeling of microstructure evolution based on the direct input from experimental 3D imaging in an acceptable time should be possible. Therefore, development of such a parallel 3D full-field CA static recrystallization (SRX) model not only for scientific, but also industrial applications, was the goal of this research.

## 2. SRX Model

The static recrystallization model selected as a case study for the current investigation was based on two main steps. The first is a nucleation step directly followed by the grain boundary migration, leading to an increase in material recrystallized fraction. To recreate the physics of the recrystallization, the developed model takes into account the influence of the two major driving forces on the process kinetics. The first controls the grain boundary migration as a function of accumulated deformation energy [32], while the second is associated with the influence of grain boundary curvature [33]. During simulation, grain boundary mobility is correlated with grain crystallographic orientations represented by misorientation [34] or Euler angles [35].

The developed CA model was based on the constitutive equation that relates the velocity of the growing grain with the mobility and the net pressure:

*v = M_g_P* (1)

where *M_g_* is the grain boundary mobility and *P* is the net pressure on the grain boundary. 

**Figure 1:**
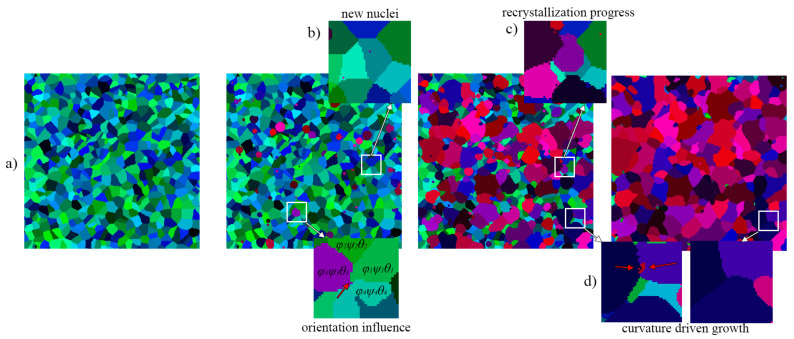
Examples of results from the CA-SRX model (**a**) initial microstructure, (**b**) nucleation stage, (**c**) recrystallization progress, and (**d**) grain growth after recrystallization (red palette represent recrystallized grains).

The energy value can be obtained directly from the EBSD investigation of a deformed sample or calculated by the numerical-based FE model of a deformation process [36]. Details on the physical nature of the developed model can be found in another author’s work [37], while examples of microstructure evolution during and after SRX are presented in Figure 1. 

The first attempt to parallelize the developed model proved its capabilities and robustness concerning the classical sequential execution [31]. However, several issues have also been identified with the developed parallelization mode that constrained further improvement in the model computational efficiency. Therefore, the current investigation is focused on developing more advanced and dedicated algorithmic solutions for model parallelization toward computing times acceptable by practical industrial calculations. Particular attention was placed on developing novel parallelization modes and communication mechanisms with their details described in the following sections.

## 3. Parallelization Modes

**Figure 2:**
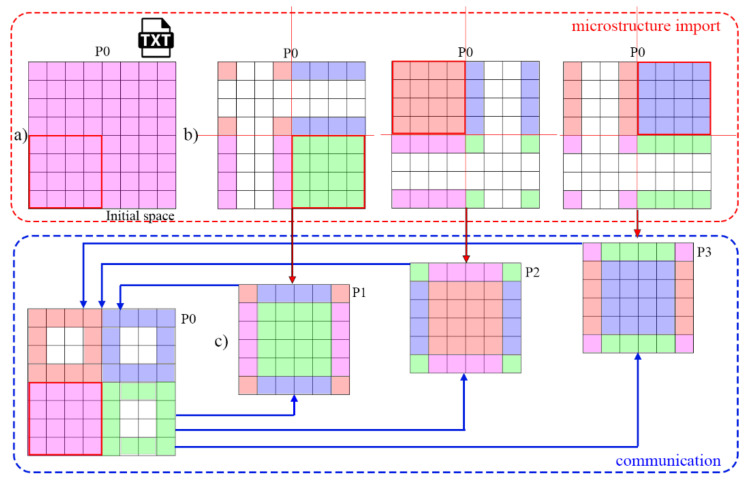
CA space preparation steps for the first parallelization scheme (mode 1). (**a**) The entire CA computational domain, (**b**) initial microstructure data loaded to the P0 process, and (**c**) information exchange within the P0 and remaining MPI processes.

The first developed parallelization solution called mode 1 is based on the classical approach with one main MPI process with pid = P0 responsible for communication and data division. The P0 process is primarily in control of a loading operation of the initial data for further calculations. The initial microstructure morphology with grain ids, crystallographic orientations, and stored deformation energy values, along with a complete set of simulation parameters, is loaded from the input .txt and .xml types of files. These data are then used to create the CA space for further calculations. The P0 is also responsible for dividing the CA space into smaller subdomains depending on the selected division scheme and an available number of processes for parallel execution. The P0 generates subsequent subdomains and an additional two-cell thickness boundary buffer that is used as a data exchange zone between processes. All these data, containing a set of appropriate microstructure information from each subdomain, are being finally distributed by the P0 between available processes: P1–PN (Figure 2a). In the approach, the P0 holds the complete information on the CA space and also performs calculations on one set of data associated with the first subdomain (Figure 2a,b). All other processes store information only about a particular received subdomain (Figure 2c). At the end of each CA algorithm iteration, all information from each subdomain and boundary cells located at subdomain edges are gathered by the P0 process (Figure 2c).

**Figure 3:**
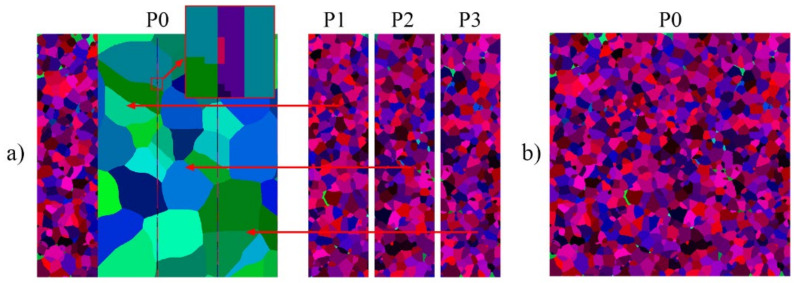
Concept of data agglomeration stage in mode 1 (**a**) data collection by the master process P0, (**b**) restored final microstructure morphology after the SRX simulation.

At the end of the entire simulation, the P0 is also responsible for the agglomeration of obtained data (Figure 3a) to allow for the complete restoration of a microstructure morphology, as seen in Figure 3b. This approach is used in the present work as a reference solution to underline the bottleneck of the communication mechanisms based on the main process P0. Limitations of the P0 process used for data distribution were also pointed out in the parallelization solution developed in [31].

**Figure 4:**
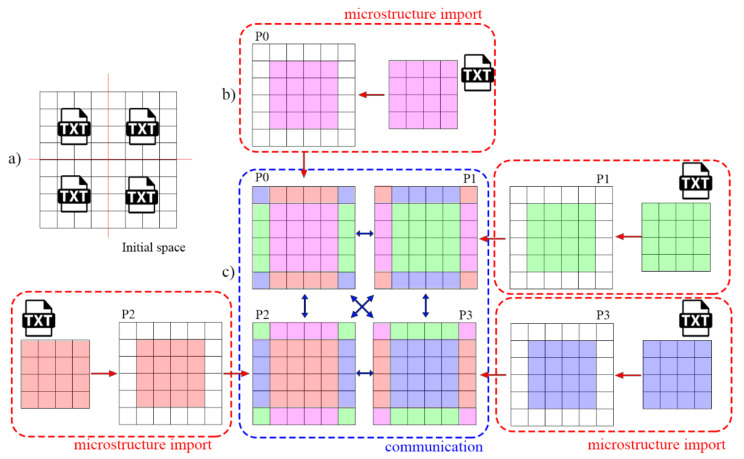
CA space preparation steps for the second parallelization scheme (mode 2). (**a**) the entire CA computational domain, (**b**) initial microstructure data loaded to each MPI process from dedicated files, and (**c**) information exchange within the boundary buffer zones for all neighboring MPI processes.

Therefore, to overcome these identified limitations, the second parallelization solution called mode 2 was developed. This solution is based on a different concept that can be classified as an equivalent processes approach, where the main process no longer controls the entire simulation. In contrast, the microstructure dataset (Figure 4a) is loaded to each MPI process independently from the dedicated .txt or .hdf5 files. Each MPI process allocates memory for the subdomain with an additional two CA cells for boundary buffer zone information exchange between neighboring processes (Figure 4b). Finally, before the first step of the simulation, each MPI process sends information from the boundary buffer cells to neighboring processes (Figure 4c).

In the developed approach, at the end of each CA SRX algorithm iteration, communication is realized only between neighboring processes via the boundary buffer zones (Figure 4c). As a result, no single process stores the entire CA space’s complete dataset, eliminating the previously mentioned bottleneck. The complete dataset is accessed at the end of the simulation by the developed postprocessing tool that agglomerates the obtained results.

However, to speed up the simulation even further, a new communication mechanism between subdomains was also developed, implemented, and evaluated.

## 4. Communication Mechanism

**Figure 5:**
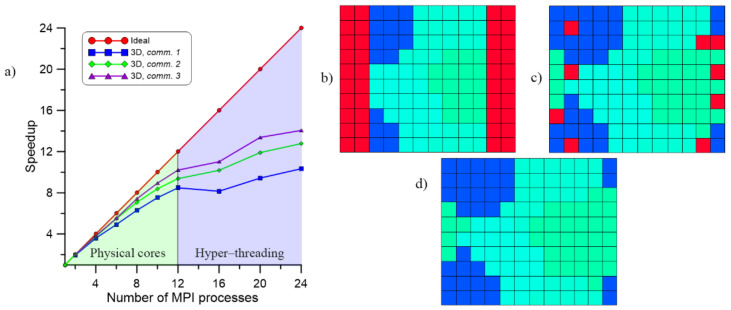
(**a**) Computation speedup with developed communication mechanisms: (**b**) comm. 1, (**c**) comm. 2, (**d**) comm. 3.

In most of the approaches that are available in the literature, the communication mechanism (comm. 1) sends information from all cells located along the boundary of the subdomain in the buffer zone to the neighboring buffer zones (Figure 5b). Even the data from unrecrystallized CA cells that have not changed during a particular iteration of the CA algorithms are exchanged, creating unnecessary computational overhead. Therefore, to minimize this overhead, a new communication mechanism (comm. 2) that sends data only from the recrystallized CA cells located within the boundary buffer zone was proposed, as seen in Figure 5c. Additionally, for comparison purposes, the unphysical SRX algorithm version without a communication mechanism (comm. 3) between subdomains was used (Figure 5d) to show the theoretical baseline and point out what would be the best possible outcome in the case of computing times. As shown in Figure 5b, the buffer region was assigned as two cells on the boundary (within the presented scenario, space was divided into *X* vertical layers). This is related to the long-range Moore neighborhood used in the curvature-driven driving force model [38]. When only energy-driven growth is considered and classical Moore neighborhood is used, the buffer zone can be reduced to a single cell on subdomain boundaries.

A comparison of computational speedup for the three mentioned communication mechanisms is presented in Figure 5. During this analysis, the CA SRX model was executed with the MPI standard on a local workstation with two Intel Xeon E5-2420v2 processors (24 cores) (Intel, Santa Clara, CA, USA) and 64 GB of RAM (Micron, Boise, ID, USA) to underline the influence of the mentioned hyper-threading. The computational domain size was set to 1000 × 1000, and simulation parameters were set to obtain 50% of the recrystallization volume fraction at the end of the process.

As can be seen in Figure 5, differences between the three investigated communication mechanisms are noticeable. The second developed mechanism based on updating only selected CA cells provided better results than full information exchange. It should be pointed out that when simulation without data exchange was considered, the ideal speedup was achieved only for two processes. Above this threshold, it slowly dropped down. Such an effect can be attributed to the available cache memory. Another interesting trend was observed above 12 MPI processes. In this case, simulation speedup was reduced significantly due to hyperthreading.

**Figure 6:**
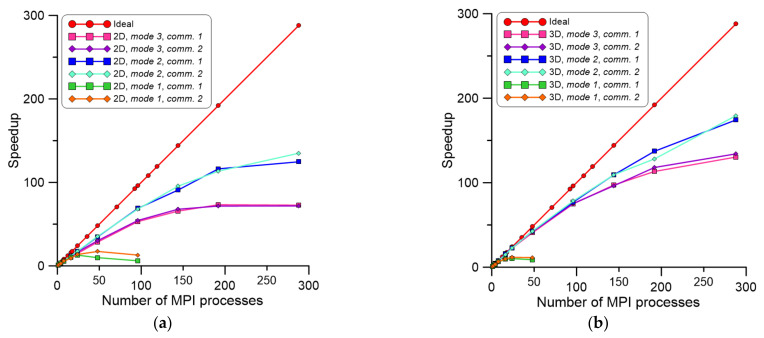
Speedup from (**a**) 2D and (**b**) 3D investigations for a different number of MPI processes.

Based on these initial results, the calculations were scaled up using the Prometheus supercomputer [39] (ACK Cyfronet AGH University, Krakow, Poland) to underline differences between different proposed parallelization schemes and extend the investigation into significantly larger CA spaces. First, 1000 × 1000 CA cells representing a physical space of 300 × 300 µm were used in 2D. All parallelization schemes were computed with a number of MPI processes raging between 1–288; multiplication of 24 processes was selected to fulfill all available processors at the provided computational nodes. The second investigation was based on 100 × 100 × 100 CA cells, representing the physical space of 30 × 30 × 30 µm. The CA space dimensions in the 2D and 3D case studies were selected in order to maintain the same number of CA cells and, therefore, the same physical size of CA cells during simulations. In addition, the number of iterations was the same in both simulated cases. The developed parallelization concepts (modes 1 and 2) were compared with the one developed in [31] (mode 3). Examples of results in the form of computation speedup are presented in Figure 6. During the simulation, times related to input/output operation and communication were gathered.

**Figure 7:**
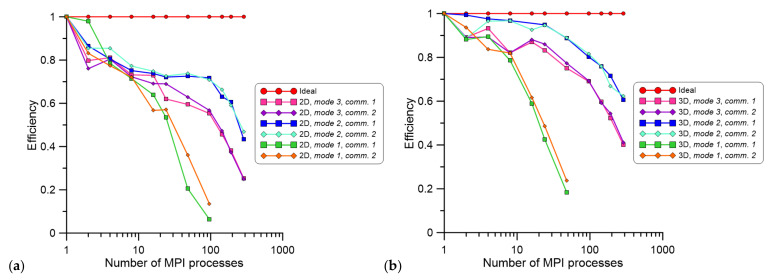
The efficiency of parallelization for (**a**) 2D and (**b**) 3D investigations for a different number of MPI processes.

As can be noted in Figure 6a, developed parallelization mode 2 delivered the best speedup of calculations. In parallelization mode 1, the speedup dramatically decreased for even a small number of processes, caused by the communication overheads. Therefore, a further increase in the number of MPI processes used for simulation was meaningless in this case, and computations were limited to 96 processes in 2D and 48 processes in 3D simulations, respectively. In the case of different communication mechanisms, both investigated approaches provided similar results. Corresponding results in the form of parallelization efficiency are presented in Figure 7.

Figure 7 highlights the fact that with an increase in the number of MPI processes, the efficiency of calculations decreases. In 2D computations (Figure 7a), this trend was faster than in the 3D ones (Figure 7b) and can be attributed to a smaller number of CA cells within a 2D Moore neighborhood that have to be evaluated during calculations. Figure 6 and Figure 7 clearly indicate that parallelization mode 1 is the least effective due to the data collection procedure performed by the P0 process. This process devotes less time to pure calculations and more time to communication and synchronization operations performed in each time step than the other two solutions. Moreover, the parallelization mode 1 and mode 3 from [31] were based on the same initial data preparation and final results collection steps, which are considered as bottlenecks. When the simulation with 192 MPI processes was analyzed for parallelization mode 3, these steps took around 10 s while the total simulation time was less than 23 s. Thus, almost half of the simulation time was due to the sequential master process calculations that involve the import of the initial microstructure from the input file and decomposition of the CA space to subsequent processes. In this case, a further increase in MPI processes is meaningless because of the sequential part of the application. Therefore, it can be summarized that parallelization mode 2 is the most effective solution for the CA SRX model. 

**Figure 8:**
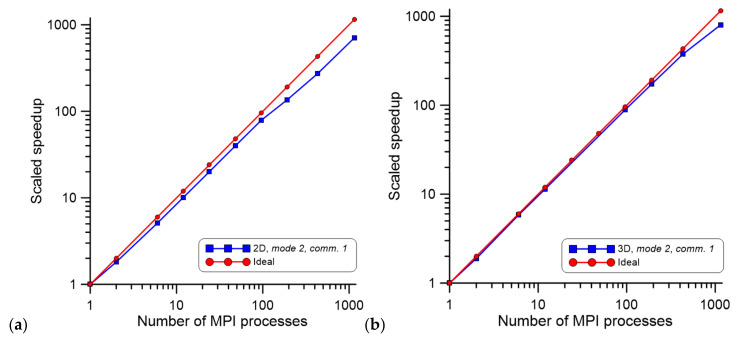
Scaled speedup for (**a**) 2D and (**b**) 3D investigations with an increasing number of MPI processes and a growing CA space size.

In the case of large computational domains, the concepts of mode 1 and mode 3 are often not applicable because of limitations in available RAM at a single computing node. Therefore, the developed parallelization mode 2 was the only solution to simulate static recrystallization progression in large computational domains with more than a billion cells in the CA spaces. To show the model scalability for larger CA spaces, the communication comm. 1 involving all buffer CA cells was used as a case study. Such a case was selected on purpose as it is the worst-case scenario, where during each simulation, all cells on domain edges are exchanged. The case study assumed that each of the MPI processes performed computations on one million CA cells. Examples of the obtained results are presented in Figure 8 in the form of scale speedup.

The presented investigation clearly shows that CA spaces with over one billion cells (1,000,000,000 CA cells) that can already represent a large volume of material can be successfully computed with the presented parallel implementation within an acceptable computational time. Therefore, with the developed approach, it is possible to accelerate computations of microstructure evolution over 700 times for a 2D scenario (Figure 8a) and over 800 times for a 3D scenario (Figure 8b), which is crucial for future practical applications of the developed model in industrial practice.

The model with such capabilities finally provides a possibility to perform calculations on high-quality input data directly acquired during the experimental investigation, again narrowing the gap between the model and actual material behavior.

## 5. Practical Case Studies

**Figure 9:**
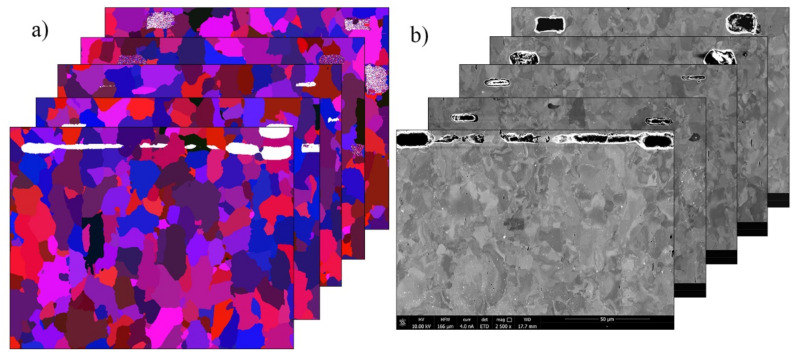
Stack of experimental 2D images of microstructure morphologies obtained via serial sectioning (**a**) EBSD and (**b**) SEM images.

Based on the above statement, the developed parallelized CA SRX model was initiated on the basis of the dataset acquired from the 3D material metallographic investigation. The approach was based on the serial sectioning procedure followed by the reconstruction process that was developed in the authors’ recent work [40]. The serial sectioning investigation was based on high-resolution scanning electron microscopy using an electron backscattered diffraction detector (SEM/EBSD) (Quanta 3D 200i, FEI, Hillsboro, OR, USA), as presented in Figure 9. Refer to [40] for the details regarding the polishing operation setup and 3D reconstruction algorithm.

**Figure 10:**
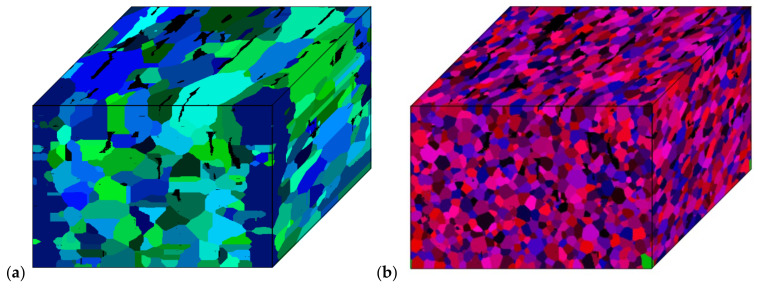
3D visualization of (**a**) initial unrecrystallized and (**b**) final recrystallized microstructure.

After the reconstruction procedure, the input data in the form of ferritic-pearlitic digital microstructure was composed of 1200 × 700 × 808 CA cells representing the material volume of 150 × 87 × 101 µm. Raw initial data were stored in .txt format (*x*, *y*, *z*, *id*, *average misorientation*) and occupied approx. 10.4 GB of storage space on a hard drive. With the use of mentioned parallelization modes 1 and 3, the incorporation of such a large amount of data into the SRX simulation would be problematic. The reconstructed digital model was then filled with the energy stored due to 20% deformation and numerically heated from 600 to 680 °C with a heating rate of 1 °C/s, and the *t_step_* was set to 0.01 s. In such a case, almost half of the simulation was under the curvature-driven growth model [34], which significantly increased simulation time. The initial material morphology with 678,720,000 CA cells (Figure 10a) was then simulated with the number of computing nodes equal to 60, which provided 1440 processes. Final material morphology after the simulation is presented in Figure 10b. As a result, the simulation time was 280 min with this setup, and approximately 280 Gb of RAM was used. However, it should also be pointed out that further time reduction is still possible within the higher number of computing nodes. 

The CA SRX simulation on such input data was not possible before developing the new parallelization and data communication schemes. Additionally, the theoretically estimated time for sequential simulation of the investigated 3D case study was 190 days, which would not be acceptable for practical application. As presented, the parallelization of the model reduced this time to approximately five hours. However, this time will be additionally extended with required data processing operations. First, preparation of initial data for computation in the distributed environment requires additional pre-processing time. Additionally, the data transfer to and from the server (above 10 GB each) should be considered. The second aspect of using supercomputers is related to sharing resources with different users. In this case, queueing time is also an additional element that slows down the overall analysis. Therefore, the overall time that was required for the presented simulation was approximately seven hours. Such computing times are still far from the possible online control of the deformation process, but they are acceptable for offline technology design. 

**Figure 11:**
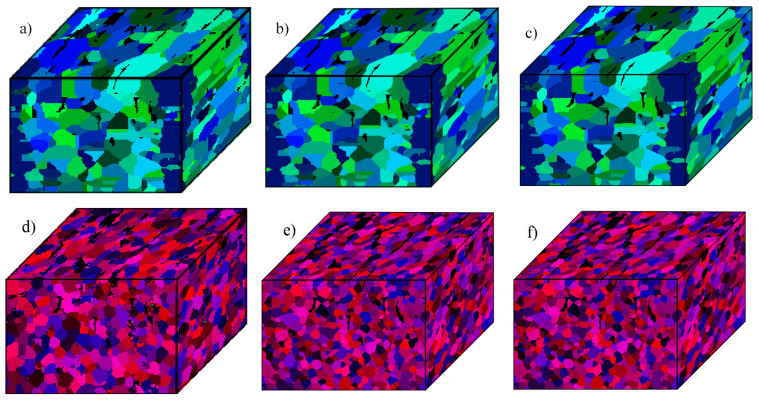
Initial material morphology after scaling to (**a**) 150 × 87 × 101, (**b**) 300 × 175 × 202, (**c**) 600 × 350 × 404 cells and corresponding results of static recrystallization simulation (**d**–**f**).

However, it should also be pointed out that there is a possibility of reducing the microstructure model’s discretization size, which will speed up calculations but will also affect the quality of grain representation, and eventually the progress of the recrystallization process. Therefore, such a model size reduction has to be performed very carefully, and the obtained results have to be validated against the full-size model. Examples of CA SRX results obtained from the initial digital microstructure (Figure 11a–c) scaled down to 150 × 87 × 101, 300 × 175 × 202, and 600 × 350 × 404 CA cells are presented in Figure 11d–f. In such cases, all simulations were computed with 24 computational nodes, which gives 576 processes used for the simulation. Such a number of processors is reasonable for typical servers in an industrial company, and all inconvenience related to the mentioned high-performance computing facilities are minimized. With such a setup, the simulation time was reduced to approx. 2, 14, and 93 min, respectively. 

As presented in Figure 11a, the grain boundaries lose their smoothness and initial shape for the microstructure with the smallest resolution. In addition, perlite islands in many regions are artificially reduced to the thickness of a few CA cells. Therefore, such a significant reduction in CA space resolution may influence the SRX predictions. However, the other two investigated resolutions provided similar results comparable to the full-size model, and could be used for metal forming process development as the computing times are reduced to a couple of minutes, even at a typical workstation. However, as mentioned, such a resolution reduction has to be done individually for each investigated material to ensure that some important microstructural features will not be artificially neglected during the simulation.

As presented, numerical simulations based on the direct input from the experimental analysis and not numerically generated artificial microstructure data are possible. They can deliver invaluable information for the industry during the process design stage. For such advanced simulations, the time frame is still quite significant; however, with the developed parallelized CA SRX model, the 3D computations can be realized within a range of minutes, while the 2D model can be executed within seconds, which provides a possibility even for an online process control.

## 6. Conclusions

Based on the presented research, it can be concluded that:

- The cellular automata method has great potential for algorithm adaptation to the distributed environment via parallelization.
- The parallelization mode 1 is clearly affected by communication overheads.
- The parallelization mode 2 is the best solution for large CA spaces but requires an additional data preparation stage.
- Parallelization is more effective with an increasing number of CA cells processed during the entire simulation; this is clearly visible for 3D scenarios.
- Communication mechanisms used during CA cell interaction in each time step directly influence model execution times.
- The speedup decreases for more than 96 and 144 MPI processes in 2D and 3D, respectively, which is related to sequential parts of the code.
- The developed model provides a possibility to obtain scaled speedup over 700 times for a 2D scenario and over 800 times for a 3D scenario, which is crucial for practical applications in industrial practice.
- With the developed solution, the computations of more than one billion CA cells are possible with reasonable time, which provides a possibility to simulate 3D microstructure models with a large number of grains with varying sizes.
- Simulations based on experimental data from the 3D reconstruction algorithm can also be undertaken with a reasonable time.

The presented approach is still open for further improvement, and therefore, the next stage of research will be focused on the preparation of a heterogeneous approach within a combination of MPI and OpenMP standards, which may be even more effective than the current concept. 

## Data Availability

The data presented in this study are available on request from the corresponding author. The data are not publicly available due to the fact that it is a part of the ongoing research.
